# Statistical factors associated with utilisation of ototoxicity monitoring services for multi-drug-resistant tuberculosis patients in the Western Cape

**DOI:** 10.4102/sajcd.v66i1.596

**Published:** 2019-01-30

**Authors:** Lebogang Ramma, Primrose T. Nhokwara, Christine Rogers

**Affiliations:** 1 Department of Health and Rehabilitation Sciences, University of Cape Town, South Africa; 2 Department of Health and Rehabilitation Sciences, Division of Communications Sciences and Disorder, University of Cape Town, South Africa; 3 Department of Health and Rehabilitation Sciences, Division of Communication Sciences and Disorders, University of Cape Town, South Africa

## Abstract

**Background:**

South Africa is a high-burden multi-drug-resistant tuberculosis (MDR-TB) country. Previously, standard MDR-TB treatment regimen in South Africa included kanamycin, an aminoglycoside, which can cause permanent hearing loss in patients. South African National Tuberculosis Control programme’s guidelines for the management of patients with MDR-TB were revised in 2011 to support outpatient-based models. This, in turn, required reorganisation of ototoxicity monitoring services to support these new models of service delivery.

**Objectives:**

The aim of this study was to determine factors associated with the utilisation of ototoxicity monitoring services for patients with MDR-TB who accessed treatment as outpatients.

**Method:**

A retrospective review of medical records of patients who attended ototoxicity monitoring clinic at a central TB hospital in Cape Town during 2012–2013 was conducted. A total of 801 medical folders were reviewed: 415 (51.8%) males and 386 (48.2%) females, median age 37 (range 7–85) years.

**Results:**

Ten per cent of patients attended all the recommended six-monthly appointments. Patients who presented with hearing loss at baseline or developed hearing loss after treatment initiation were more likely to attend their appointments. Patients were also more likely to attend their appointments if a baseline audiometric assessment was conducted within 1 month of MDR-TB treatment initiation.

**Conclusion:**

This study revealed that outpatient-based ototoxicity monitoring services were largely underutilised by patients. Development of hearing loss and prompt determination of a baseline audiogram were associated with a higher likelihood of attendance of ototoxicity monitoring appointments. Therefore, utilisation of outpatient-based ototoxicity monitoring services is likely to be improved by identifying patients early and monitoring them closely.

## Introduction

South Africa is ranked third among the 22 high-burden tuberculosis (TB) countries. The latest (2017) World Health Organisation (WHO) report estimated that TB incidence in South Africa is approximately 781 (95% CI, 543–1060) or more cases per 100 000 people, thus making it a country with one of the highest incidence rates of TB in the world (WHO, 2017). South Africa also has the second highest number of patients diagnosed with either MDR-TB or rifampicin-resistant tuberculosis (RR-TB) globally (Churchyard et al., 2014). The WHO estimated that the annual RR-TB or MDR-TB incidence rate in South Africa is about 34 (95% CI, 22–45) per 100 000 people or 3.4% (95% CI, 2.5% – 4.3%) and 7.1% (95% CI, 4.8% – 9.5%) among new and retreatment TB cases, respectively. Latest data showed at least 19 073 laboratory confirmed cases of RR-TB or MDR-TB during 2016 (WHO, 2017).

The WHO’s recommended second-line drugs to be used for treating MDR-TB still include amikacin and kanamycin, both aminoglycoside antibiotics (WHO, 2016). Until 2018, the South African MDR-TB treatment regimen also included kanamycin, an aminoglycoside antibiotic with many significant side effects (Department of Health [DOH], 2013; Duggal & Sarkar, 2007). One of the most common adverse effects resulting from treatment with kanamycin is permanent sensorineural hearing loss (SNHL). Incidence of permanent SNHL has been reported to be up to 57% in a group of South African MDR-TB patients and may vary in severity from slight to profound (Harris, Peer, & Fagan, 2012; Ramma & Ibekwe, 2012). Hearing loss, if it affects frequencies that are important for speech perception, is associated with marked communication difficulties (Arlinger, 2003). If left untreated, these communication difficulties may result in social isolation, feelings of exclusion, reduction in social activity, depression, anxiety and negatively impact on the socio-emotional well-being of those affected (Manrique-Huarte et al., 2016).

In recognition of this high risk of developing hearing loss, and to avoid potentially negative consequences that could result from MDR-TB treatment-induced hearing loss, South African treatment guidelines for MDR-TB require that patients’ hearing thresholds be monitored (i.e. ototoxicity monitoring) when they are treated with a regimen that includes kanamycin (Department of Health, 2013). The Health Professions Council of South Africa’s (HPCSA) guidelines on the management of patients who are treated with drugs that include ototoxic medications also recommend ototoxicity monitoring for patients treated with drugs such as kanamycin (HPCSA, 2018). Ototoxicity monitoring involves scheduled, repeated audiometric assessment of patients’ hearing thresholds over time. Changes in hearing status allow review of drug regimens and management of any hearing loss that may result because of treatment (Dinnes, Hewison, Altman, & Deeks, 2012; HPCSA, 2018).

Prior to 2011, national policy guidelines in South Africa on the management of MDR-TB mandated that all MDR-TB patients be initiated on treatment only after they had been admitted to the country’s few specialised TB hospitals (Department of Health, 2013). It was relatively easy to provide ototoxicity monitoring services for these patients, as they were institutionalised and, therefore, available. However, in 2011, policy guidelines for the management of patients with drug-resistant TB were revised to support outpatient-based MDR-TB treatment services (including ototoxicity monitoring). Outpatient-based MDR-TB treatment meant that patients could now access services as outpatients in a facility nearest to them, instead of centralised TB hospitals. Decentralisation was expected to lead to better access to ototoxicity monitoring by patients because of close proximity of services to those who need them. Proximity to facilities has been reported elsewhere to be an important factor in increasing health seeking and utilisation of services (Al-Mandhari et al., 2008; LaVella et al., 2004). Some studies have reported on other factors besides geographical proximity that can influence utilisation of service. For instance, Vaidya et al. (2012) reported on the influence of sex and likelihood of utilising health care services; women generally tend to have higher service utilisation rates when compared with men. This study therefore aimed to describe utilisation patterns and determine factors associated with the utilisation of ototoxicity monitoring services when provided as outpatient services.

## Materials and methods

This was a descriptive, retrospective review of medical records of all patients who accessed outpatient-based ototoxicity monitoring services during January 2012 to March 2013 at Brooklyn Chest TB Hospital (BCH) in Cape Town.

At the time of this study (2012–2013), patients in the Western Cape were already receiving their daily MDR-TB treatment (Directly Observed Treatment [DOT]) at their nearest primary health care clinics. Patients who lived closer to BCH were usually referred by their facilities to the hospital for ototoxicity monitoring. In addition, some patients were required to come to BCH once a month for a follow-up appointment with a specialist MDR-TB doctor who was based there. Hospital protocol required audiometric assessment (ototoxicity monitoring) at the start of patients’ MDR-TB treatment and during their monthly follow-up appointments with the doctor.

Ototoxicity monitoring protocol at the hospital was as follows: Identification of patients by the medical and nursing staff during MDR-TB treatment initiation and referral to audiology for baseline audiogram. Baseline audiometric assessments included otoscopic examination, tympanometry and pure tone audiometry (air conduction [AC]: 250 Hz – 8000 Hz and bone conduction [BC]: 250 Hz – 4000 Hz). Patients were monitored once a month (over a 6-month period) at a time when they came for their monthly follow-up appointment with the doctor. Monthly audiometric monitoring tests included otoscopic examination and pure tone AC from 250 Hz to 8000 Hz.

Patients who showed a significant hearing threshold shift (STS) following treatment initiation underwent a comprehensive audiometric evaluation (tests administered at baseline) and were referred back to the doctor for medical management. Monitoring intensity in patients who showed a significant STS increased to biweekly instead of monthly. Patients who developed a disabling hearing loss were managed according to the hospital’s aural rehabilitation protocol (amplification, communication repair strategies and auditory training as indicated).

An STS posttreatment initiation was defined according to the American Speech and Hearing Association’s (ASHA) 1994 criteria; the presence or absence of hearing loss was defined according to Margolis and Saly (2007) classification (i.e. pure tone average [PTA]: 0.5 kHz, 1 kHz and 2 kHz] ≤25 dB HL = no hearing loss, PTA: 0.5 kHz, 1 kHz and 2 kHz ≥26 dB HL = hearing loss) and disabling hearing loss was defined as a hearing loss >40 dB HL (PTA: 0.5 kHz, 1 kHz, 2 kHz and 4 kHz) in the better ear (adults) (WHO, 2014).

Descriptive statistics (means and medians) were used to describe central tendencies in the data. Log-binomial regression (generalised linear modelling) (*p* < 0.05) was used to determine variables associated with the likelihood of attending a scheduled appointment for ototoxicity monitoring. Data were analysed using the statistical programme Stata (release 13) (Stata Corp, 2013).

### Ethical consideration

Ethical clearance was sought and obtained from the University of Cape Town’s Faculty of Health Sciences Health Research Ethics Committee prior to the commencement of data collection (HREC REF: 604/2012). Permission was granted from the Provincial Department of Health and the management of the hospital facility before conducting the study. Only records of patients who had a confirmed diagnosis of MDR-TB, and attended ototoxicity monitoring services at least once during the review period, were included for review. Patients’ records with missing information (e.g. drug regimen) and records of XDR-TB patients were excluded. Relevant data from patients’ medical folders, demographics, MDR-TB treatment and audiometric data were recorded into a password-protected Excel spreadsheet for analysis at a later stage.

## Results

### Patients’ description

A total of 801 patient medical folders were accessed for review in this study. There was an equal proportion of males and females; most of the patients were from within the Cape Town metropolitan area; the majority were treated with kanamycin (as the only aminoglycoside) (Table 1).

**TABLE 1:** Summary of patient description (*n* = 801).

Variable	*n*	%	Years	Range
**Sex**				
Male	415	52	-	-
Female	386	48	-	-
Median age	-	-	37	7–85
**Proximity of referral area to facility**				
<20 km: City Bowl, Southern suburbs, Northern suburbs	223	28	-	-
20 km – 100 km: Atlantic Seaboard, Cape Flats, Helderberg, South Peninsula and West Coast	546	68	-	-
>100 km: Cape Winelands, Eden and Central Karoo	31	4	-	-
**Treatment regimen**				
Kanamycin	689	86	-	-
Kanamycin and streptomycin	15	2	-	-
Streptomycin	30	4	-	-
Other aminoglycosides	66	8	-	-
**Duration between treatment initiation and initial hearing test**				
<1 month	176	22	-	-
1–3 months	521	65	-	-
4–6 months	80	10	-	-
>6 months	24	3	-	-
**Hearing thresholds**				
No hearing loss (PTA 0.5 kHz – 2 kHz ≤ 25 dB HL)	397	50	-	-
Hearing loss (PTA 0.5 kHz – 2 kHz ≥ 26 dB HL)	404	50	-	-
Significant threshold shift	315	64	-	-
No significant threshold shift	175	36	-	-
Disabling hearing loss	139	17	-	-
No disabling hearing loss	661	83	-	-

dB HL, decibels hearing level; PTA, pure tone average.

**FIGURE 1:**
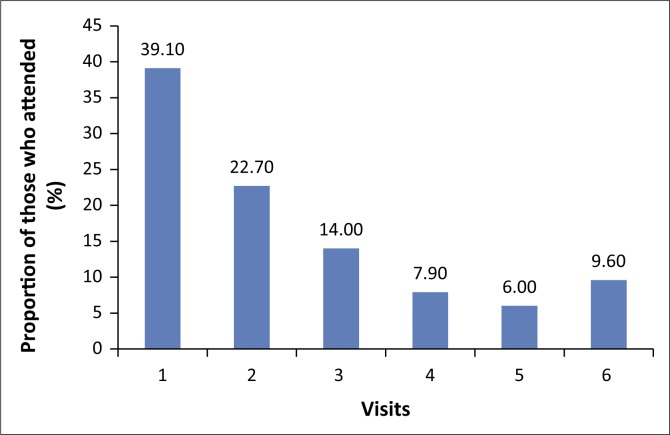
Pattern of attendance of scheduled ototoxicity monitoring appointments.

An analysis of the pattern of attendance of scheduled appointments was also conducted, and it was found that only 10% of the patients attended all of the recommended six appointments for ototoxicity monitoring that they were scheduled to attend (Figure 1).

Relative risk estimation by log-binomial regression (*p* < 0.05) was used to investigate the association between different variables and likelihood of attending a scheduled appointment for ototoxicity monitoring. It was found that the longer the time taken to conduct baseline audiometry assessment after treatment initiation, the less likely the patients were to utilise the services. In terms of hearing thresholds, compared to those with a hearing loss at the time of initial hearing assessment, those with normal hearing were 30% less likely to attend (RR 0.7 [95% CI, 0.6–0.9], *p* = 0.02) a scheduled visit. Those who developed hearing loss during the course of treatment were 60% more likely to attend when compared with those who did not develop hearing loss (RR 1.6 [95% CI, 1.3–2.2], *p* = 0.0). Patients with a disabling hearing loss were 120% times more likely to attend their scheduled appointment for ototoxicity monitoring than those without a disabling hearing loss (RR 2.2 [95% CI, 1.7–2.8], *p* = 0.0). Age, sex, treatment regimen and proximity of referral area to the facility where services were accessed were not associated with whether the patient will attend their appointment or not (see Table 2).

**TABLE 2:** Variables associated with regular attendance of scheduled ototoxicity monitoring appointments (i.e. 4–6 visits).

Variable	Sub-variable	N	% 4–6 visits	*p*	RR	95% CI
Proximity of referral area to facility	<20 km	223	25	-	1.0	Ref.
	20 km – 100 km	546	24	0.5	0.3	0.2–0.7
	>100 km	31	39	0.1	0.5	0.3–0.9
Treatment regimen	Kanamycin	689	24	-	1.0	Ref.
	Kanamycin and streptomycin	15	27	0.8	1.1	0.5–2.6
	Streptomycin	30	13	0.2	0.5	0.2–1.4
	Other aminoglycosides	66	27	0.5	1.1	0.8–1.7
Waiting period	<1 month	124	38	-	1.0	Ref.
	0–3 months	379	26	0.007	0.7	0.5–0.9
	4–6 months	59	15	0.005	0.4	0.2–0.8
	>6 months	18	22	0.2	0.6	0.2–1.4
Hearing status	Hearing loss	404	28	-	1.0	Ref.
	No hearing loss	397	20	0,02	0.7	0.6–0.9
Significant threshold shift	No	175	27	-	1.0	Ref.
	Yes	315	45	0.00	1.6	1.3–2.2
Disabling hearing loss	No	661	20	-	1.0	Ref.
	Yes	139	44	0.00	2.2	1.7–2.8
Age	7–29	239	23	-	1.0	Ref.
	30–39	232	21	0.5	0.8	0.6–1.2
	40–49	189	26	0.5	1.1	0.8–1.6
	50–71	141	28	0.2	1.2	0.9–1.7
Sex	Male	415	23	-	1.0	Ref.
	Female	386	25	0.6	1.0	0.8–1.4

RR, rifampicin-resistant.

## Discussion

This study showed that an overwhelming majority (90%) of patients did not attend the recommended number of ototoxicity monitoring visits when services were provided on an outpatient basis. Patients were more likely to utilise the services if a baseline audiometric assessment was conducted within a month of initiating treatment, they had a pre-existing hearing loss (PTA_0.5–2 kHz_ ≥26 dB HL) at baseline or they developed hearing loss during the course of treatment. Interestingly, a close proximity between the patient’s area of residence and the facility was not associated with a higher likelihood of attending scheduled appointments.

Only about 10% (9.6%) of the patients whose medical folders were reviewed attended all of the recommended six ototoxicity monitoring appointments as per protocol at the facility where services were provided. At the time of this study, patients on MDR-TB treatment were at the highest risk of ototoxicity-induced hearing loss during the first 4–6 months (intensive phase) of their treatment when they were undergoing daily kanamycin injections (TB Alliance, 2015; WHO, 2017). Therefore, a protocol at the facility required that these patients undergo monthly audiometric assessment during the first 6 months of treatment to monitor their hearing thresholds. Duggal and Sarkar (2007) recommended that these patients should ideally be monitored throughout the course of treatment and then for up to 6 months posttreatment with ototoxic aminoglycoside because of the propensity for ototoxic hearing loss to continue to progress even after the use of these drugs has been withdrawn or discontinued. It is apparent that an opportunity has been missed to actively manage patients with possible ototoxic hearing loss.

Patients were more likely to attend their scheduled ototoxicity monitoring visits if a baseline audiometric assessment was conducted before, or shortly after they initiated MDR-TB treatment. The recently published HPCSA ototoxicity monitoring guidelines recommend that a baseline assessment be conducted before or within 72 hours of treatment with ototoxic aminoglycoside such as kanamycin (HPCSA, 2018). Baseline assessments assist in defining any pre-existing hearing loss, which is a risk factor for further deterioration of hearing thresholds as a result of aminoglycoside exposure. A baseline assessment also enables comparison with subsequent tests to evaluate whether ototoxicity has occurred (Durrant et al., 2009). Therefore, it is important to identify patients early, conduct baseline assessments and provide them with information that will empower them to use ototoxicity monitoring services.

It was also found that patients who presented with hearing loss (i.e. PTA_0.5–2 kHz_ ≥26 dB HL) at baseline; patients who developed hearing loss during treatment and those with disabling hearing loss were more likely to use ototoxicity monitoring services when compared to patients with normal hearing thresholds. This was consistent with reports from previous studies, which reported that patients who perceive themselves to have a hearing loss are likely to seek intervention for their problem (Meyer & Hickson, 2012; Ramma & Sebothoma, 2017). Garstecki and Erler (1998) also reported that individuals who had a severe degree of hearing loss were likely to adhere to ototoxicity monitoring services, thus supporting the notion that hearing loss, especially when severe, is a motivating factor for adherence to recommended ototoxicity monitoring services.

A study by Vaidya et al. (2012) suggested that there is a difference between males and females when it comes to health care service utilisation, with a higher uptake in women. However, there was no significant difference in the patterns of utilisation of the services between men and women in the current study. Differences in service utilisation could be because of the fact that for some types of health care services, women have higher utilisation rates than men (Gerritsen & Devillé, 2009). Ototoxicity monitoring, therefore, could be an example of the type of services where there is no significant difference in service utilisation between sexes.

An unexpected finding in this study was the lack of association between a close proximity of patients’ place of residence (based on area of referral) to the health facility and the likelihood of using outpatient-based ototoxicity monitoring services. Close proximity facilitates utilisation of health care services (Al-Mandhari et al., 2008; LaVella et al., 2004), while conversely distance may act as a barrier when services are remote from where the individuals reside (Fujita et al., 2017; Nteta et al., 2010). It was, therefore, expected that patients who lived closer to health care facility (i.e. better geographical access) would show higher utilisation rates for ototoxicity monitoring services. However, in this study it was found that proximity to health facility was not necessarily associated with a higher utilisation of ototoxicity monitoring services. These findings seem to suggest that simply making services available closer to patients may not be sufficient to ensure high uptake of services. Other interventions such as active campaigns to inform patients (Zamawe, Banda, & Dube, 2016) about the value of ototoxicity monitoring services and improving the quality (Audo, Ferguson, & Njoroge, 2005) of these services may be required to increase patients’ utilisation. Thus, quality of the service, comprehensive follow-up and patient empowerment are important considerations for future roll-out of outpatient-based ototoxicity audiology services.

This was one of the first studies conducted in South Africa to systematically evaluate the utilisation of outpatient-based ototoxicity monitoring for MDR-TB patients. Therefore, it gave insights that can inform future decentralised service delivery models for audiology services in general. However, the study also had several limitations related to its design. Firstly, the nature of the study was a retrospective record review. Secondly, because it was a descriptive quantitative study, the reasons for underutilisation could not be ascertained from the records. Thirdly, the study was conducted during the early phases of decentralisation of MDR-TB treatment, and the health system was still undergoing a series of modifications to enable this new model of service provision (i.e. outpatient MDR-TB treatment). However, positive developments have occurred in the past 5 years to improve access to ototoxicity monitoring at primary care facilities. These include employing more audiologists dedicated to the provision of ototoxicity monitoring for MDR-TB patients, a national roll-out of a portable audiometry programme to enable audiometric assessments at primary health care facilities as well as involvement of non-audiology personnel (e.g. MDR-TB Nursing Sisters) in audiometric assessment for ototoxicity monitoring (Mngemane, Ndjeka, & Ramma, 2017). More importantly, a recent (2018) announcement to introduce a kanamycin-free treatment regimen for MDR-TB (Medecins Sans Frontieres, 2018) by the South African National Department of Health will change how ototoxicity monitoring occurs (if any) in these patients. It is, therefore, highly likely that these interventions have significantly changed utilisation patterns for ototoxicity monitoring services under a decentralised model of MDR-TB treatment model.

## Conclusion

The findings of this study showed that centrally located outpatient-based ototoxicity monitoring services for MDR-TB patients were significantly underutilised by patients. The services also lacked responsiveness, as shown by most of the patients in this study getting their baseline audiological assessment more than a month after they initiated treatment. The duration between treatment initiation and baseline audiometry assessment, pre-existing hearing loss at baseline audiological assessment, development of ototoxic hearing loss during treatment and the development of disabling hearing loss were all associated with a higher likelihood of adhering to the recommended ototoxicity monitoring plan. Proximity to services (or facility) was not necessarily associated with higher likelihood of utilisation of services. It is, therefore, recommended that interventions aimed at improving uptake of outpatient-based ototoxicity monitoring services must aim to conduct a baseline assessment before or within the recommended 72-h posttreatment initiation.
